# Sailors and the Risk of Asbestos-Related Cancer

**DOI:** 10.3390/ijerph18168417

**Published:** 2021-08-09

**Authors:** Richard A. Lemen, Philip J. Landrigan

**Affiliations:** 1 United States Public Health Service (Retired), Rockville, MD 20852, USA; 2 Rollins School of Public Health, Emory University, Atlanta, GA 30333, USA; 3 National Institute for Occupational Safety and Health (Retired), Atlanta, GA 30333, USA; 4 Collegium Ramazzini, 41012 Carpi, Italy; 5 Program for Global Public Health and the Common Good, Boston College, Boston, MA 02467, USA; 6 Medical Corps, United States Naval Reserve (Retired), Washington, DC 20351, USA

**Keywords:** asbestos, cancer, lung, mesothelioma, exposure, permissible exposure limit (PEL), pleural, seamen, seafarers

## Abstract

Sailors have long been known to experience high rates of injury, disease, and premature death. Many studies have shown asbestos-related diseases among shipyard workers, but few have examined the epidemiology of asbestos-related disease and death among asbestos-exposed sailors serving on ships at sea. Chrysotile and amphibole asbestos were used extensively in ship construction for insulation, joiner bulkhead systems, pipe coverings, boilers, machinery parts, bulkhead panels, and many other uses, and asbestos-containing ships are still in service. Sailors are at high risk of exposure to shipboard asbestos, because unlike shipyard workers and other occupationally exposed groups, sailors both work and live at their worksite, making asbestos standards and permissible exposure limits (PELs). based on an 8-h workday inadequate to protect their health elevated risks of mesothelioma and other asbestos-related cancers have been observed among sailors through epidemiologic studies. We review these studies here.

## 1. Introduction 

Earlier findings of asbestos-related diseases among shipyard workers [1] should have provided warning about the potential dangers of asbestos-related diseases to sailors. While shipyard workers are exposed to high levels of asbestos for defined work periods, usually 8–10 h, merchant seamen live and work 24 h per day in the shipboard environment. Aboard ships at sea, asbestos fibers can be released at any hour of the day or night due to ship repairs during a voyage or as the result of vibrations that release airborne asbestos fibers into the many confined spaces aboard ships where sailors work and live. These asbestos fibers can result in inhalation exposure.

Airborne asbestos fibers can travel long distances from their point of release to result in inhalation exposure. Reitze et al. reported in 1972 that asbestos fibers were found in concentrations of 70 fibers per cc 3 m away from the source and 46 fibers per cc up to 25 m away from their source [2]. Once asbestos fibers are disturbed and/or released into the environment, they can continuously be re-entrained into the air in confined spaces until they are removed or contained [3]. These finding have clear implications for the exposure of sailors in confined spaces at sea.

## 2. Methods

We searched for asbestos-related disease (ARD) publications in the National Library of Medicine database ‘PubMed’ and the National Institute for Occupational Safety and Health database ‘NIOSHTIC-2′ by using the following keywords: e.g., ‘asbestos’, ‘mesothelioma’, ‘lung cancer’, ‘seamen’, ‘sailor’, ‘maritime’, ‘navy’, and ‘merchant marine’. PubMed was selected because it is the largest database with free access for both biomedical and life-science literature. NIOSHTIC-2 was similarly selected because it contains the largest U.S. database specifically for occupational disease and safety. We entered our keywords under the respective search fields of the database. The inclusion criteria were original scientific articles or reviews by year of publication. Analytical and descriptive epidemiologic studies were considered in the review, which included cohort, case-control, and cross-sectional studies, as well as case reports and case series. In addition to the two major databases searched, we searched additional sources including government databases such as records maintained by the U.S. Maritime Administration. Additionally, references not found in the original search were identified among review of the cited references found in the original articles identified in the original database searches. The medical conditions of interest were mesothelioma, lung cancer, and interstitial and pleural abnormalities.

We categorized our findings into the following six subject areas: (1) cohort study, (2) case-control study, (3) cross-sectional studies, (4) case reports, (5) case series, and (6) miscellaneous. There were three premises to assign each article to relevant subject categories. First, each article was published in only one peer-reviewed journal. Second, each journal article was assigned to one or more of the relevant subject categories. Third, each article was reviewed by the authors for relevance to the study objective of identifying risk of ARD to sailors. Primarily English language articles were included, but other foreign language articles were included for which translations could be obtained.

## 3. Asbestos Use in Ships

**Figure 1:**
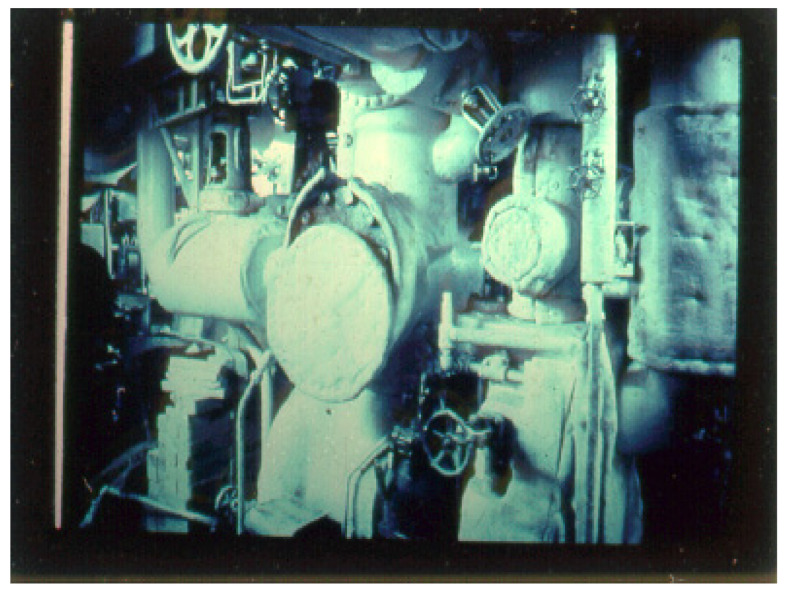
Asbestos insulation in the engine room of a ship. Photo courtesy of Irving J. Selikoff, MD Archives.

Asbestos-containing products on ships include joiner bulkhead systems in living spaces, insulation on both hot water and steam piping, inside and outside of boilers and tanks, and in machinery parts and other products used on board ships (Figure 1) [4]. The asbestos-containing bulkhead panels are often erroneously called fiberboard, insulating board, or wallboard, among several names. When asbestos was first used in bulkhead panels on ships the core mix was routinely made of asbestos fiber and diatomaceous or hydrous calcium silicate held together with inorganic binders having a density of approximately 38 pounds per cubic foot, which is much lighter than most alternate materials [4].

Asbestos-containing dust can be released from bulkhead panels not only when installed, but also during repairs or any period where the core of the panel is disturbed. Asbestos in machinery casings, block insulation, asbestos cement, and lagging, pre-formed asbestos insulation, including on steam pipes, flanges and valves, and vinyl asbestos tile for decking and flooring, are all sources of asbestos found on board ships. When the vessel is at sea, flaking and cracking from vibrations due to the ship’s motions can release fibers from any of these sources. Although there is no conclusive evidence, it may be of concern that structural corrosion caused by salt water and air could facilitate the release of the anti-corrosive asbestos from its supporting matrix, e.g., asbestos-cement products that are highly corrosive to salt. This issue should be further evaluated.

**Figure 2:**
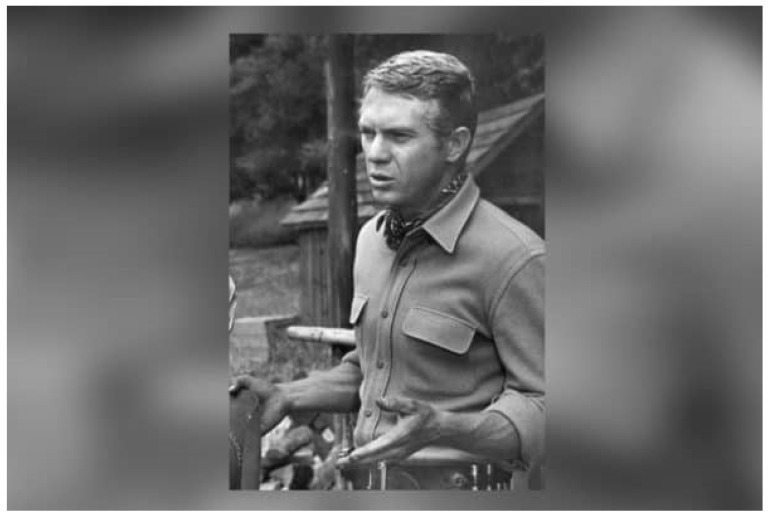
The well-known American actor, Steve McQueen, died of mesothelioma in 1980 at the age of 50. McQueen’s first exposure to asbestos likely occurred during his service in the U.S. Marine Corps from 1947 to 1950. Through his service, McQueen spent time working onboard naval ships and in the shipyards.

Additionally, it is not unusual for merchant seamen to make in-route repairs on asbestos-containing equipment. Measurements aboard ships at sea have documented the release of asbestos fibers, and concentrations in the range of 0.001–0.152 fibers/cc greater than 5 microns in length have been recorded. While some of these levels exceed the current US OSHA 8-h permissible exposure limit (PEL) for airborne asbestos of 0.1 fibers/cc, they should not be relative for the sailor who lives and works in this environment 24 h per day 7 days per week. Compare these to samples taken during asbestos removal operations on ships, like those measured from the Long Beach Naval Shipyard, which averaged from 40 fibers/cc to as high as 150 fibers/cc, which serve as an indicator to the possibility of much higher concentrations if similar repairs on ships occur while at sea [5]. The highest concentrations, on two U.S. flag ships while at sea, occurred during anchor winch brake box cleaning using compressed air for dust removal, which generated upward of 70 fibers/cc of asbestos-containing dust, while a routine brake operation recorded concentrations in the 2.1 fibers/cc range. Clean up by dry sweeping, after repairs, measured fiber concentrations as high as 3.4 fibers/cc. Welding resulted in upwards of 5 fibers/cc asbestos and background fiber concentrations as high as 1 fibers/cc were recorded, thought to result from the ship motion and vibration. The findings were collected on two consecutive nights leading the authors to suggest these concentrations may have existed during the past 24 h [6,7,8,9]. Studies of the James River Reserve Fleet in 1982 found that 24 of 168 ships recorded airborne asbestos fibers in concentrations of 0.4–0.10 fibers/cc, though samples from the engine room had levels 3 times higher. Analysis by transmission electronic microscope (TEM) found both chrysotile and amphibole asbestos [7] (Figure 2).

A review of bulk samples collected between 1978 and 1992 from merchant ships funded by “*Various owners and/or operators of merchant vessels*” and subsequently published in 2008, reported that 28 of 42 ships sampled contained asbestos-containing insulation. The highest concentrations were found in the engine room areas, but not while any active disturbance or work was being done that would further disturb any asbestos-containing materials [10]. These findings were much lower than those collected by the U.S. Maritime Administration.

Samples taken on board ships at sea cannot be compared for safety to a PEL, which is based on an 8-h workday. Thus, these findings reflect a much lower risk than a sailor’s true exposure to asbestos, since they both work and live on the ship 24 h per day 7 days per week. For example, an average worker inhales 6–8 million cubic centimeters of air per an 8-h working shift. Therefore, at a standard of 2 million asbestos fibers larger than 5 microns in length per cubic meter of air would mean somewhere in the range of inhaling 16 million “*permissible*” fibers. However, for sailors’ onboard a ship, this does not reflect their true exposure to asbestos, since they never leave their workplace. Additionally, it should be recognized that these measurements miss many inhaled fibers since those less than 5 microns in length are not counted using the phase contrast microscope (PCM) for comparison to existing PEL-based standards. Additionally, it is a misnomer to presume that fibers shorter than 5 microns in length are not a part of the health hazard since the 5-micron cut-off length was selected, not based on health risk, but rather as a result of the limits and the economically accepted use of the PCM method of analysis, which is used for regulatory purposes.

Using the PCM method, the relative proportion of airborne fibers longer than 5 microns may vary from 1% to 50% of the total asbestos in a particular air sample. In fact, Dement and Wallingford, from NIOSH, found the PCM was a poor predictor of total fiber exposures of all lengths. They found in typical occupational environments, fibers less than 5 microns outnumber the longer fibers by a factor of 10 or more [11,12]. As this relates to asbestos-induced diseases, it has been found that most asbestos fibers in mesothelial tissues were shorter than 5 microns in length [13,14,15]. Another study of lower lobe lung cancer found fibers below 5 microns in length (3–5 µm) compared to greater than 5 µm gave similar results [16]. OSHA’s risk assessment, as published in the Federal Register, showed that while reducing the PEL to the 0.1 f/cc level would reduce disease, it would not eliminate the significant risk of adverse health effects and that exposures at this level were still estimated to pose a lifetime risk of death from asbestos-related cancer of 3.4 per 1000 workers and a 20-year exposure risk of 2.3 per 1000 workers [17,18].

As stated, on 12 February 1980, in a testimony before the Subcommittee on Coast Guard and Navigation of the House Committee on Merchant Marine and Fisheries, one of the authors (R.A.L.) stated: “*During World War II, 4.5 million workers are estimated to have worked in shipyards. Currently, there are some 120,000 shipyard workers employed in about 450 establishments in the United States. Several epidemiologic studies have been conducted of persons involved in the building, maintenance, and repair of seagoing vessels. Each of the studies has shown an excess of asbestos-associated diseases, including asbestosis, lung cancer, and mesothelioma. In some cases, asbestos-induced changes in the lung were detected in the X-rays of over 50 percent of the workforce. Mortality among shipyard workers has been much greater than that expected when compared with control populations. This excess has mainly been accounted for by diseases associated with asbestos exposure.*” This statement reflects the high magnitude of asbestos-related disease (ARD) resulting from asbestos used in ships. Based on these findings, maintenance at sea could generate exposures in much higher fiber ranges. Studies of industrial workers in asbestos trades have shown excessive disease and death for workers exposed at much lower levels than are reported during these rip out operations. To date, scientists have been unable to determine a dosage level below which asbestos will not cause disease [19] which is still true today [1]. In conclusion to the Congressional testimony, one of the authors (R.A.L.) stated: “*Control of exposures to seafaring persons should include, as the U.S. Navy has now required, that no new ships contain asbestos and that maintenance, repair, and asbestos removal operations be controlled to the greatest extent possible. This would involve assuring appropriate protective clothing and work practices for those directly involved and keeping non-protected persons away from areas in the ship where asbestos exposure can occur*” [19]. If recommendations, as those presented to the U.S. Congress in 1980, are followed, most of the disease patterns described next could be reduced and/or prevented. Regulations addressing these recommendations are now coming into effect and efforts to ban asbestos are gaining support.

Studies have been conducted of sailors involved in the maintenance, repair, and operation of seagoing vessels. Most of these studies have shown an excess of asbestos-associated diseases, including asbestosis, lung cancer, and mesothelioma. Routine maintenance at sea can result in significant exposures to asbestos as respirable size fibers are released and become airborne, thus placing sailors at an increased risk of developing asbestos-related diseases, especially asbestos-induced cancer, e.g., lung cancer and mesothelioma. In fact, the United States Maritime Commission studies found that “*Long after the vessel had been at sea, flaking and cracking due to ships motions and vibration and motion are suspected of releasing asbestos into the surrounding space*” [4]. Thus, the hazards from asbestos exposures are not confined to shipbuilders only, but to a vessel’s crew while operating the ship. As stated in the Maritime Administration report, “*In the course of a voyage it is not unusual for crewmen to repair pipe, pipe flanges, or valve leaks and this generally means a tear-down situation. We must assume then that machinery and piping asbestos insulation as we have described, affect not only the shipyard worker, but the crew as well under a variety of conditions*” [4]. As determined by the Maritime Administration, government-subsidized ships, contracted prior to 1973 “*showed no discrimination against the use of asbestos insulation and lagging for piping and machinery, nor in the area of joiner bulkheading*” [4]. While it is thought many uses of asbestos ceased on such government-subsidized ships, asbestos was not eliminated entirely since “*asbestos insulating cement (used for lagging of machinery casings) and asbestos lagging cloth were still specified*.” However, between the years 1975 and 1978, “*Even though asbestos materials were not listed as options, neither were they prohibited, and some asbestos-based materials have found their way into the ships where yard practices permitted*” [4]. Since vessel delivery is about two years, this would mean a two-year lag after contract specifics changed, leading the Maritime Administration to believe most ships built under the government-subsidized program and delivered after 1979–1980 would essentially be free of asbestos. Therefore, for U.S. government-subsidized ships constructed prior to 1980, it would mean “*… the majority of vessels in the U.S. flag fleet at present have asbestos insulating materials in the machinery spaces as well as in the accommodations areas…*” [4]. While this explains the consequences for specifications related to government-subsidized ships it does not address the situation for non-government-subsidized ships. Thus, to determine whether a ship contains asbestos would necessitate a review of ship specifications and testing.

## 4. Epidemiological Findings of Disease among Sailors

In 1918, the first report of radiological change among seamen exposed to asbestos was reported in a marine fireman [20].

A study of 5041 licensed marine engineers reported an overall 12% prevalence of abnormalities in chest roentgenograms with a steady progression in prevalence of 2% for those with union membership between 0 and 5 years, then rising to 27% for those having membership greater than 35 years [21]. Even 12% is high when compared to the 4.4% of prevalence of pleural abnormalities found in a group of United Kingdom dockyards workers in 1966 [22], and in dockyard workers surviving until 1977 this prevalence had risen to 12.4% [23]. Jones et al. concluded that “*The presence of abundant asbestos in the working environment, the frequent need for marine engineers to remove and reapply insulating materials, the high fiber counts shown to result from these operations, and the roentgenographic survey results presented in this article support a hypothesis that marine engineers have received significant asbestos exposure*” [21]. The authors point to anecdotal evidence of asbestosis and mesothelioma found in marine engineers as indicating sufficient exposure to asbestos to constitute a risk for lung cancer as well. Greenberg, when looking at a subset of radiographs on a mixture of subjects having either PA radiographs or some having both PA and lateral radiographs, found that “*the prevalence of pleural abnormalities detected overall was 19.6%. Postmortem studies demonstrated the presence of pleural thickening to be in excess of that detected radiographically.*” Thus, “*The stated prevalence of pleural abnormality in any population studied radiographically will be an understatement of the true prevalence*” [24]. Greenberg continues to report seamen have experienced excess mortality from cancer for over the past 100 years and that a preliminary mortality analysis of a small population of merchant seamen reported two cases of malignant mesothelioma, and the United Kingdom’s national mesothelioma register reported 28 cases in seamen [24].

A study of radiological abnormalities among 3324 long-term United States merchant marine seamen taken between 1985 and 1987 found the highest prevalence of asbestotic changes among those who served in the engine department (391/920; 42.5%) when compared to other departments (deck 301/820, 36.6%; steward 278/981, 28.4%), or multiple departments (167/541, 30.9%). The abnormalities increased with years worked. A third had parenchymal or pleural abnormalities with pleural changes being the most predominant [25]. The parenchymal changes (small opacities) were as follows: 0/0 = 67.9%; 0/1 = 15.3%; 1/0 = 9.3%; 1/1 = 5.7%; 1/2 = 0.8%; and 2/1 and higher = 0.9%. These 3324 readings were divided into 3 categories: (1) absence of pleural fibrosis, 2929 (88%); (2) circumscribed pleural fibrosis, 325 (10%); and (3) diffuse pleural fibrosis, 70 (2%) [25]. The abnormalities increased with the duration of shipboard exposures. The authors of this U.S. study reported “*Data are available indicating that merchant marine seamen in the United Kingdom have significant adverse mortality experience, including an approximate doubling of total deaths (all causes), total cancer deaths, and deaths from lung cancer (for categories deck, engine room hands, bargemen, light tenders, and boatmen; deck, engineering and radio officers, and pilots, ship; and foremen, ships, lighters, and other vessels)*” [26,27].

In a study of seamen over 50 years of age in Japan, they reported that a higher prevalence of asbestos-related pleural plaques on X-rays occurred in 10% of 90 engineers compared to 2% in 126 deckmen and stewards, which was significantly different between the two groups. These findings when compared to a control group of workers in steam repair shops for steam locomotives found 13.7% of 146 workers with pleural plaques, though none were found in 100 clerks of the same company, prompting the authors to suggest “*Older seamen or retired seamen should be followed up carefully in every ocean-going country*” [28].

The United States Public Health Service (USPHS) studied mortality patterns of U.S. merchant seamen, who were patients of eight U.S. Public Health Service hospitals between 1973 and 1978. Overall, in the eight hospitals, seamen accounted for 19.3% of respiratory cancers as compared to 9.3% in non-seamen. In one USPHS hospital, the Staten Island, NY hospital, 55% of seamen died of cancer compared to 27.8% of non-seamen. In this same hospital, 20.2% of seamen had respiratory cancers compared to only 7.6% among non-seamen. When contrasted to mortality in the U.S. short-stay hospital system, the eight USPHS hospital cancer-related deaths, particularly those of respiratory cancer, exceeded those of heart disease. This study found “*Mortality patterns, for seamen and non-seamen overall, are quite similar except for deaths associated with respiratory cancer. For this cancer, the proportion of seamen deaths, nearly one out of five, is double that of non-seamen.*” The authors point out that environmental asbestos exposure has been well documented in seamen [4,21,29,30,31,32]. Unfortunately, the USPHS hospitals, specifically charged in providing medical care for merchant seamen, were dismantled in 1981, making further epidemiologic studies, while necessary, more difficult to pursue.

Mortality among 2208 Italian seamen enrolled in a study between 1936 and 1975 and followed through 1989 observed that lung cancer deaths were significantly increased, while the overall cohort had fewer deaths than expected. When dividing the cohort into two groups, one of 948 reporting at least one sailing experience and 1260 reporting no sailings, excess lung cancer was observed only in the group with at least one sailing experience (standardized mortality ratio (SMR) = 1.71; 95% CI: 1.15–2.44). The trend in SMRs for lung cancer increased with the duration of employment. The authors state “*Asbestos may be implicated, since at least one confirmed case of mesothelioma* (SMR = 5.87)*, a* “*sentinel health event*” *of asbestos exposure, occurred in the cohort.*” While smoking was not controlled for “*It should be noted, however, that: (i) other smoking-related diseases, such as ischemic heart diseases and bronchitis, are not elevated in cohort A (Steenland et al., 1984); and (ii) the results obtained by the internal comparison of cohort A with cohort B, together with the positive trend in lung cancer mortality with duration of employment, are unlikely to be explained by smoking habits (Siemiatycki et al., 1988)*” [33,34,35].

A retrospective cohort mortality study of Danish seamen, between 1970 and 1985, found overall high mortality among all groups of seamen. The highest rates of mortality were found among young and unmarried seamen. For cancer in the respiratory system, mortality rate ratios (MRR) were high in both engine officers (MRR = 1.90 (95% CI: 1.39–2.60)) and machine crew (MRR = 2.47 (95% CI: 1.59–3.82)). There was some concern that these respiratory cancers could be related to smoking; however, as the authors point out “… *it is striking that we did not find a corresponding excess mortality among deck officers and crew, from whom we would expect almost the same smoking habits.*” Other studies of marine engineers and machinists did not show excesses of these other non-respiratory cancers but did find lung cancers could be explained by exposures to asbestos, oil mist, and exhaustion fumes from the engine rooms. The authors point out that other confounders could relate to the high number of deaths for which there was no information on cause and lack of exposure measurements for possible carcinogens [36].

In a group of 141 retired Greek merchant seamen, researchers found 58 (41%) had one or more asbestos-related pleural abnormalities. The abnormalities in the Greek study were not limited to marine engineers, but were also related to other occupations aboard ships and provide modest evidence that the pulmonary function may result from low-level exposure to asbestos. “*The results of this study give evidence that mariners may have asbestos-related disease many years from onset of employment aboard merchant marine vessels*” [37]. These findings were somewhat higher than those found by the American marine engineers’ study by Jones et al. in 1984 [21]. In another study of Greek merchant seamen, two mesotheliomas, one in a marine engineer having 35 years of service and the other in a deck department seaman having 25 years of service, were reported. The authors point to the case in the deck department as an indicator that “*… exposure to relatively low concentrations of asbestos fibers, which may occur as these products become friable*” and that “*The case of mesothelioma in a mariner of the deck department is of special interest in the context of the risk posed by exposure to low levels of asbestos.*” In conclusion the study reported “*… there is no exposure level known without risk*” [38].

Lung cancer was found significantly elevated among Icelandic marine engineers (SMR 175; *p* < 0.05, one tailed) and machinists (SMR 2.34; *p* < 0.05, one tailed) studied between 1951 and 1982. No excess was found for overall cancers in this cohort and a special survey of smoking status showed cigarette smoking was less common among in the members of the cohort than in the general population of Reykjavik. “*These results support the suggestion that the increased mortality of lung cancer in the study group had a causal relationship to occupational exposure, particularly to asbestos exposure*” [39]. In another study of Icelandic seamen, between 1958 and 1986 that followed from 1966 to 1989, found a significantly elevated overall lung cancer excess of 113 deaths compared to the 72.32 expected (SMR = 1.56; 95% CI: 1.30–1.87). The authors concluded that “*The excess lung cancer is in agreement with the results from and Italian study of seamen* [36]*, the U.S. Study* [31]*, and the Norwegian study* [40]”. The rates for lung cancer increased with latency, while the smoking habits of ordinary seamen in Reykjavik area were found to be above average, making it uncertain if the excess lung cancer was related to occupation only. The fact that no mesothelioma was identified was not surprising to the authors since “*…pleural mesotheliomas are frequently unrecognized and may not be reported on death certificates.*” The authors “*…conclude that the excess mortality from unknown causes indicates a special situation among seamen and might be due to hazardous working conditions or behavioural* [sp.] *or lifestyle factors.*” In a follow-up study, Rafnsson and Gunnarsdóttir reported on cancer incidence of Icelandic seamen rather than mortality, as in their first study. This study consisted of 27,884 fishermen and sailors from the merchant fleet who were members of a pension fund for seamen during 1958–1986. There were 758 cancers vs. 688.43 expected. Lung cancers accounted for 132 cases vs. 82.06 expected (standardized incidence ratio (SIR) = 1.61; 95% CI: 1.36–1.91). While the authors explained that it was difficult to point to the most likely explanation for the excess lung cancer risk, they did conclude it could be related to smoking or perhaps asbestos exposure, but since cancer of the esophagus and bladder were low, as other authors have reported [34], this raises doubt to the role of cigarette smoking. In conclusion, the authors say by preforming an incidence study as compared to a cohort mortality study their findings indicate that “*…lung cancer seems to be associated with the occupation of seamen*” [41].

Cancer incidence among 30,940 male and 11,529 female Finnish seafarers from 1967 to 1992 reported that the engine crew had a significant excess of lung cancer occurring 10 years since the first employment (SIR = 1.5; 95% CI: 1.1–2.0), but that did not increase with more exposure, although smoking-related cancers did not show any apparent excess. Among the male ship deck crew, lung cancer was slightly elevated but not significantly (SIR = 1.2; 95% CI: 0.9–1.6). Mesothelioma occurred in both the engine room crew (2) and deck crew (1). In female seafarers, the overall incidence of lung cancer was significantly elevated (61 observed (obs) vs. 24 expected (exp), SIR = 2.6; 95% CI: 2.0–3.3), and after 20 years since the first employment, it continued to increase (22 obs vs. 7 exp, SIR =3.1; 95% CI 1.9–4.7). Because cancers of the mouth, pharynx, and larynx were also increased among female seafarers, both smoking and alcohol could not be ruled out as confounders; however, the authors concluded “*…it is likely that occupational asbestos exposure among seafarers causes some excess mesothelioma…*” [42].

A follow-up study of 15 million people from five Nordic countries classified by occupation and cancer incidence found seamen among the highest group having significantly elevated SIRs (SIR = 1.27; 95% CI: 1.19–1.35). Mesothelioma showed the largest differences between occupations. Seamen in Denmark and Norway had elevated cancer risks. Lung cancer among male seamen were among those with the highest risk (*n* = 3583; SIR = 1.62; 95% CI: 1.57–1.68) with the highest SIR for histological subtypes of lung cancer being adenocarcinoma (SIR = 1.71), closely followed by small cell (SIR = 1.58), squamous cell (SIR = 1.58), and other (SIR = 1.63) cancers. Smoking could not be ruled out as a confounder, nor could exposure to asbestos in the machine room, nor polyaromatic hydrocarbons and oil mist as risk factors for the elevated lung cancers, though seamen had a high risk for mesothelioma (obs = 143; SIR = 2.18; 95% CI: 1.85–2.56), which the authors pointed to asbestos as the “*overwhelming cause*”. In total, 14 of 53 occupational groups studied involved exposure to asbestos [43]. In another study of 893,264 Norwegian men, followed between 1971 and 1991, reported seamen, after adjusting for smoking, had an SIR for lung cancer of 1.25 (95% CI: 1.2–1.4) [44].

A cohort study of 6603 marine engineers followed from 1955 to 1998 observed 810 cancers when 794 were expected (SIR = 1.0; 95% CI: 1.0–1.1), as well as an excess of lung cancers (124 obs vs 100.98 exp SIR = 1.2; 95% CI: 1.0–1.5), and that after a 40-year latency period, lung cancer increased to an SIR of 1.4 (95% CI: 1.2–1.8). Additionally, four mesotheliomas were observed among those graduating prior to 1961. Smoking habits of the engineers were similar by age with that of the reference general population and could not be explained solely by confounding alone or due to differences in smoking habits. The authors concluded that there is still an increased risk of lung cancer in the shorter latency interval, though not significantly; thus, lung cancer risk in the future could not be ruled out. The authors say “*In conclusion, the increased risk for mesothelioma points to previous asbestos exposure. The excess found for lung cancer could also be related to asbestos exposure*” [45].

Baksaas et al., in another Norwegian study, found excess mortality from lung cancer among all groups of seamen [40].

In a clinicopathological correlation study of 1445 cases of mesothelioma, merchant marine seamen and U.S. Navy had the second highest number of mesotheliomas of any occupational category after shipbuilding. Further, the authors reported 4 of 37 (11%) merchant marine and U.S. Navy mesothelioma patients also had asbestosis [46].

In the United States, a multi-center case-control study of hospital patients with lung cancer (1980–1989) observed merchant seamen and deckhands with a non-significantly elevated odds ratio (OR) of 2.1 (95% CI: 0.7–6.2) for lung cancer [47], while a Norwegian study reported a significant excess OR of 6.97 (95% CI: 2.34–20.76) for lung cancer among seamen on oil tankers and when working as a mate on all tankers with an OR of 15.20 (95% CI: 4.80–48.20). Smoking could not totally explain the high risk in lung cancer for those working as a mate, nor could other chemical exposures be entirely ruled out [48].

In a study of mesothelioma in the Trieste Province, Italy between 1968 and 1987, 19 cases (11.2%) were reported for various trades of seamen in the Navy and merchant marine including machinist (9), Navy official (4), cook (2), electrician (2), cabin-boy (1), and steward (1). Among these cases, 57% had asbestos bodies identified from lung tissue sections. This study adds further evidence of risk from asbestos-related disease among maritime trades. Engine room seamen had a particularly high risk as did other trades among seamen with asbestos-related abnormalities, e.g., pleuropulmonary alterations being indicative of asbestos exposures among all trades of seamen [49]. In the Monfalcone area of Italy, asbestos exposure accounted for 92 cases of malignant mesothelioma followed between October 1979 and April 1992 [50]. Pleural plaques in a 79-year-old man were found on X-ray 6 years prior to his lung cancer. He had been a farmer most of his life, but at age 26, he had served on a battle cruiser for one year during World War II as a boiler man, his only known exposures to asbestos. He was a 26 pack a year smoker and had 3348 asbestos bodies per gram of dry lung tissue [51].

A case-control study among male Finnish seafarers looked at cancer among 30,940 men who had worked aboard Finnish ships for any period between 1960 and 1980, and the study found lung cancer among engine crew members significantly increasing with the duration of employment after 3 years (OR = 1.68; 95% CI: 1.17–2.41), while deck officers did not experience an increase. However, deck crew on icebreakers did have a significantly increased risk of lung cancer after greater or equal to 20 years since their first employment (OR = 3.41; 95% CI: 1.23–9.49) and mesothelioma among the engine crew was significantly increased (OR = 9.75; 95% CI: 1.88–50.6) [52].

In terms of work-related cancer in the Nordic countries, seamen were among the occupations with the highest SIR for all cancers. Overall seamen from four Nordic countries had a significant excess risk of lung cancer (obs = 1137, SIR = 1.51; 95% CI: 1.43–1.60) [53]. “*The excess risk of pleural cancer among seamen is probably not only due to those working in machine rooms, since also deck personnel seem also to have a high risk of mesothelioma*” [42].

Roggli et al. reported that patients with mesothelioma and asbestosis had higher pulmonary fiber burdens than those without asbestosis, a finding the authors say is consistent with epidemiologic evidence of mesothelioma occurring in individuals “*…with brief, low-level, or indirect exposures to asbestos*” [54].

A Swedish study on time trends and occupational risk factors for pleural mesothelioma followed men between 1961 and1998 and found that the risk for seamen was significantly increased (obs = 11, SIR = 2.83; 95% CI: 1.41–5.09), though a later follow-up between 1970 and 1998 did not find this (obs = 4; SIR = 1.98, 95% CI: 0.51–5.12). Mesothelioma for seamen, recorded in 1960, showed the highest SIR of 7.43 (*n* = 10, 95% CI: 3.54–13.72) [55]. These findings could reflect either a declining use of asbestos in Swedish ships and/or better prevention techniques for Swedish sailors.

Studying Danish seafarers, Kaerlev et al. reported non-officers had a 1.5 times higher lung cancer risk than officers. Increased risks for lung cancer presented by longest job category found the highest SIR among maintenance crew (SIR = 4.1, 95% CI: 2.1–7.4), followed by engine room crew (SIR = 2.3, 95% CI: 1.6–3.3), catering crew (SIR = 2.1, 95% CI: 1.3–3.2), deck crew (SIR = 1.9, 95% CI: 1.4–2.4), galley crew (SIR = 1.7, 95% CI: 1.0–2.9), and navigation officers (SIR = 1.3, 95% CI: 1.0–1.6). Sorted by type of vessel, dry cargo vessels, including container ships, showed officers with a lower lung cancer risk (obs = 29, SIR = 1.5; 95% CI: 1.0–2.1) than non-officers (obs = 45, SIR = 2.6; 95% CI: 1.9–3.5). In passenger ships, lung cancers were also less in officers (obs = 23, SIR = 1.0, 95% CI: 0.6–1.5) than non-officers (obs = 51, SIR = 1.6, 95% CI: 1.2–2.1). In tankers and gas tankers, non-officers had excess lung cancer (obs = 26, SIR = 3.0, 95% CI: 2.0–4.5) as it was for non-officers in smaller ships and supply ships (SIR =1.9; 95% CI: 1.2–2.8). Overall, for all ships, both officers and non-officers had elevated SIRs. Unfortunately, smoking was not controlled for nor were occupational exposures. Because of the young age of the cohort along with the 1975 ban on asbestos in Denmark, it was not surprising that no mesotheliomas were found [56].

Although, in an extended follow-up to the Danish cohort of merchant seafarers, the overall cancer incidence increased in both male and female seafarers (SIR = 1.19; 95% CI: 1.15–1.23 and SIR = 1.14; 95% CI: 1.07–1.22, respectively). Male seafarers who worked in areas with asbestos exposure showed a significantly increased risk of mesothelioma. Both males and females had excess lung, bronchus, and trachea cancers (men: obs = 687, SIR = 1.51; 95% CI: 1.40–1.63 and women: obs = 101, SIR = 1.37; 95% CI: 1.13–1.67). The highest risk of mesotheliomas occurred among machinists and engine room crew (obs = 11; SIR = 2.31; 95% CI: 1.28–4.17). The observed mean latency among these seafarers was very long, approaching 50 years. This study when compared to the previous analyses was older and had a longer follow-up period (29 years). The authors attribute the majority of overall cancers, among Danish seafarers, to be lifestyle related, and conclude that occupational hazards, such as asbestos and UV radiation, affect the cancer pattern as well [57].

Among 28,300 servicemen in the Royal Norwegian Navy, asbestos was thought to account for exposure to some 11,500 crew members serving aboard ships between 1950 and 1987. Significant excesses of mesothelioma were observed in engine room crews (SIR = 6.23; 95% CI: 2.51–12.8) and lung cancer was nearly 20% higher than expected among both engine crews and non-engine crews. Land-based crews serving less than 2 years on board a ship each had lower lung cancer rates than expected (SIR = 0.77; 95% CI: 0.64–0.92). Overall, a 65% increase in mesothelioma was observed among marine engine crews that the authors attribute as an indicator of the presence or absence of asbestos exposure, but with no consistent explanation for the variation in incidence of other asbestos-related cancers. Asbestos removal was done on all Norwegian Naval ships in the 1980s. The latency for the excess mesothelioma ranged between 28 and 48 years, consistent with asbestos-induced mesothelial tumors [58].

Overall, seamen and marine engineers are exposed to asbestos used in gaskets, pipes, valves, and machinery. With ship motion, vibration, or during maintenance, these asbestos fibers can be released into areas of the ship and put seamen at risk of asbestos-related disease. Such risks include higher prevalence of pleural abnormalities or significant excess of mesothelioma, having SIRs ranging between 1.83 and 4.8, related to the duration of exposure, and lung cancers with SIRs ranging between 1.10 and 1.62. Filon et al. reported that mesothelioma ranged around 2.5% in seamen and marine engineers, which was not the case from the epidemiological analysis of fishermen [59].

A more recent study of five different Nordic countries, published in 2020, included 81,741 seafarers and 66,926 fishermen observed between 1961 and 2005, and found lung cancer in excess among both seafarers (SIR = 1,62; 95% CI: 1.57–1.68) and fishermen (SIR = 1.16; 95% CI: 1.12–1.21). However, for mesothelioma, only seafarers had a significant excess (SIR = 2.17; 95% CI: 1.83–2.56), which was “*particularly clear in the later periods of follow-up.*” Because of the gradual and late bans of asbestos on ships through Safety of Life at Sea (SOLAS) conventions and with the full ban on new ships not occurring until 2011, many ships still contain limited amounts of asbestos, meaning seafarers in this cohort may have been exposed occupationally to asbestos [60].

A study funded by law firms representing defendants in cases of asbestos litigation, and dealing with the historical state of knowledge on the health risks of asbestos posed to seamen serving on merchant ships, concluded that “*starting in the 1990s, findings of modest increases in lung cancer and/or mesothelioma in some epidemiology studies of seamen led some authors to propose that a causal link between shipboard exposures and asbestos-related disease existed. Limitations in these studies, however, together with mostly unremarkable measures of airborne asbestos on merchant ships, preclude definitive conclusions in this regard*” [61].

## 5. Conclusions

The main finding of this review is that virtually all published epidemiologic studies of sailors report elevated morbidity and mortality from mesothelioma, lung cancer, and other asbestos-related diseases, thus indicating that sailors are at high risk of asbestos-related disease. Current asbestos measurements and protective standards based on an 8-h workday and a 5-day work week underestimate sailors’ true risk of asbestos exposure, since sailors both work and live at their workplace 24 h per day, 7 days a week and are at risk of exposure to asbestos throughout this time.

Some of the studies we cite have limitations, as they differ in their methodologic details, and they are not always able to reconstruct sailors’ precise level of past exposure to asbestos. Nonetheless, and despite these limitations, the consistent finding of a high risk of asbestos-related cancers in virtually all epidemiologic studies undertaken among sailors provides a powerful indication that asbestos is a pervasive cause of malignant disease and premature death in this population. Consistency of findings across a wide range of studies undertaken by different investigators in different populations is a powerful indicator of the validity of the findings. As epidemiologist Prof. David Savitz notes “*…studies can compensate for one another’s weaknesses.*” In other words, one study’s weakness may be compensated in another’s strength, which can be “*…central to the interpretation of a series of studies, and therefore warrants closer examination*” [62].

It is widely accepted by the scientific community and by national and international public health authorities that asbestos causes increased rates of mesothelioma, lung cancer, laryngeal cancer, ovarian cancer, and other asbestos-related diseases. Therefore, it is not surprising that there is consistent evidence from multiple epidemiologic studies of elevated risk for asbestos-related diseases among sailors. There can be no doubt of a causal relationship between asbestos exposure among sailors and a subsequent increased risk of asbestos-related diseases.

## Data Availability

Not applicable.
