# Correction: Lemen, R.A.; Landrigan, P.J. Sailors and the Risk of Asbestos-Related Cancer. *Int. J. Environ. Res. Public Health* 2021, *18*, 8417

**DOI:** 10.3390/ijerph182111518

**Published:** 2021-11-02

**Authors:** Richard A. Lemen, Philip J. Landrigan

**Affiliations:** 1 United States Public Health Service (Retired), Rockville, MD 20852, USA; 2 Rollins School of Public Health, Emory University, Atlanta, GA 30333, USA; 3 National Institute for Occupational Safety and Health (Retired), Atlanta, GA 30333, USA; 4 Collegium Ramazzini, 41012 Carpi, Italy; 5 Program for Global Public Health and the Common Good, Boston College, Boston, MA 02467, USA; 6 Medical Corps, United States Naval Reserve (Retired), Washington, DC 20351, USA

## Text Correction

There was an error in the original article [1]. The error concerned the unit of air when referring to the standard of 2 million asbestos fibers longer than 5 microns in length.

A correction has been made to Section 3, paragraph 5: “Therefore, at a standard of 2 million asbestos fibers larger than 5 microns in length per cubic meter of air would mean somewhere in the range of inhaling 16 million “*permissible*” fibers.”

The authors apologize for any inconvenience caused, and state that the scientific conclusions are unaffected. The original article has been updated.

## References

[1] Lemen R.A., Landrigan P.J. (2021). Sailors and the Risk of Asbestos-Related Cancer. Int. J. Environ. Res. Public Health.

